# Induction of effective and antigen-specific antitumour immunity by a liposomal ErbB2/HER2 peptide-based vaccination construct

**DOI:** 10.1038/sj.bjc.6602526

**Published:** 2005-04-05

**Authors:** A Roth, F Rohrbach, R Weth, B Frisch, F Schuber, W S Wels

**Affiliations:** 1 Laboratoire de Chimie Bioorganique - UMR 7514 CNRS/ULP, Faculté de Pharmacie, 74 route du Rhin, 67400 Illkirch, France; 2 Chemotherapeutisches Forschungsinstitut Georg-Speyer-Haus, Paul-Ehrlich-Strasse 42-44, 60596 Frankfurt am Main, Germany

**Keywords:** cancer vaccines, ErbB2, HER2, liposomes, lipopeptide, renal cell carcinoma

## Abstract

Efficient delivery of tumour-associated antigens to appropriate cellular compartments of antigen-presenting cells is of prime importance for the induction of potent, cell-mediated antitumour immune responses. We have designed novel multivalent liposomal constructs that co-deliver the p63–71 cytotoxic T Lymphocyte epitope derived from human ErbB2 (HER2), and HA307–319, a T-helper (Th) epitope derived from influenza haemagglutinin. Both peptides were conjugated to the surface of liposomes via a Pam_3_CSS anchor, a synthetic lipopeptide with potent adjuvant activity. In a murine model system, vaccination with these constructs completely protected BALB/c mice from subsequent s.c. challenge with ErbB2-expressing, but not ErbB2-negative, murine renal carcinoma (Renca) cells, indicating the induction of potent, antigen-specific immune responses. I.v. re-challenge of tumour-free animals 2 months after the first tumour cell inoculation did not result in the formation of lung tumour nodules, suggesting that long-lasting, systemic immunity had been induced. While still protecting the majority of vaccinated mice, a liposomal construct lacking the Th epitope was less effective than the diepitope construct, also correlating with a lower number of CD8^+^ IFN-*γ*^+^ T-cells identified upon *ex vivo* peptide restimulation of splenocytes from vaccinated animals. Importantly, in a therapeutic setting treatment with the liposomal vaccines resulted in cures in the majority of tumour-bearing mice and delayed tumour growth in the remaining ones. Our results demonstrate that liposomal constructs which combine Tc and Th peptide antigens and lipopeptide adjuvants can induce efficient, antigen-specific antitumour immunity, and represent promising synthetic delivery systems for the design of specific antitumour vaccines.

The identification of tumour-associated antigens (TAA) and TAA peptide epitopes recognised by cytotoxic T lymphocytes (CTLs) in the context of major histocompatibility complex (MHC) class I molecules has provided the basis for the development of specific cancer immunotherapies aimed at the activation of potent T-cell-mediated immune responses against cancer cells (Van Der Bruggen *et al*, 2002; Finn, 2003). Peptides representing Tc epitopes of TAAs can be used to prepare structurally defined synthetic vaccines and their use, which may be particularly advantageous, has been validated in model systems. However, although injection of antigenic peptides together with adjuvants like GM-CSF, or application of peptide-pulsed dendritic cells in some cases, resulted in antigen-specific T-cell responses in cancer patients, so far clinical responses have only rarely been observed (Jäger *et al*, 2002; Finn, 2003). Owing to the limited success of such peptide-based vaccines in the clinic, attempts are being made to improve vaccine formulations by including, for example, synthetic vectors and adjuvants to enhance *in vivo* tumour antigen uptake and presentation by professional antigen-presenting cells (APCs) such as dendritic cells (DCs), and optimise the induction of T-cell responses (Sheikh *et al*, 2000; Tartour *et al*, 2000).

The aim of our study was the design of a peptide-based cancer vaccine construct consisting of well-characterised synthetic components able to generate a powerful cell-mediated immune response against tumour cells. We have developed a novel liposomal formulation for *in vivo* delivery of a Tc peptide epitope of the shared TAA ErbB2 (HER2/*neu)* as a clinically relevant model antigen (Disis *et al*, 1994). The ErbB2 molecule is an EGF-receptor-related receptor tyrosine kinase that belongs to the class of unmodified self-antigens. Gene amplification and ErbB2 overexpression have been observed in many human tumours of epithelial origin and have been linked with cancer development and progression (Olayioye *et al*, 2000). Several HLA-A2 restricted ErbB2 peptide epitopes have been defined, including epitopes recognised by *ex vivo* stimulated CTLs on ovarian, breast, renal cell carcinoma, gastric cancer and melanoma cells (Fisk *et al*, 1995; Peoples *et al*, 1995; Brossart *et al*, 1998; Kono *et al*, 1998; Rongcun *et al*, 1999). While it could be shown that vaccination of cancer patients with ErbB2 peptide vaccines can induce or enhance ErbB2-specific immune responses, this did not result in clinical responses (Knutson *et al*, 2001).

The use of liposomes as potential carriers for vaccines has been extensively explored (Alving *et al*, 1995). Among their many advantages, these vesicles are characterised by a marked absence of toxicity and a low intrinsic immunogenicity. In addition, liposomes, because of their architecture/size and composition, offer a wide range of options for the design of effective synthetic peptide formulations to elicit both humoral and cell-mediated immune responses (Bergers *et al*, 1995). Thus, the antigens can be attached to the outer surface, encapsulated free within the internal aqueous space or incorporated within the lipid bilayers of the liposomes. The same liposomes can also play the role of carriers for various adjuvants such as monophosphoryl lipid A, lipopeptides, CpG or cytokines operative in the activation of APCs (Singh and O'Hagan, 2002). An important advantage of the use of these vesicles as vaccine vectors resides also in: (i) their ability to be efficiently endocytosed by APCs such as immature dendritic cells (Chikh and Schutze-Redelmeier, 2002), the key players in antitumour host responses (Whiteside and Odoux, 2004), and (ii) the finding that Tc antigens associated with liposomes can be efficiently processed by these APCs for MHC class-I-dependent cross-presentation to CTLs (Alving and Wassef, 1994; Rothwell *et al*, 2000; Machy *et al*, 2002). Multiepitope vesicles can also be conveniently made (Boeckler *et al*, 1999), that can carry different Tc peptide epitopes, or combinations of Tc epitopes with T-helper (Th) epitopes; this latter strategy is particularly appealing, because CD4^+^ T-cell responses are believed to play a key role in tumour immunity such as, for example, *in vivo* CD8^+^ T-cell response priming (Wang and Livingstone, 2003) and/or memory generation (Bourgeois and Tanchot, 2003). Finally, with respect to the priming of class I-restricted CTLs, it was shown previously that lipidated Tc peptide epitopes (e.g. conjugated to palmitoic acid) become highly efficient activators of CTLs (Schild *et al*, 1991; Gahery-Segard *et al*, 2000). Such lipid tails provide the covalently linked peptide moiety with intrinsic adjuvant activity (Vitiello *et al*, 1995), and allow the anchoring of the peptide to liposomes. A similar result can be achieved by conjugating Tc epitopes to lipopeptides such as *S*-[2,3-bis(palmitoyloxy)-(*2RS*)-propyl]-*N*-palmitoyl-(*R*)-cysteinyl-(*S*)-seryl-(*S*)-serine (Pam_3_CSS) (Deres *et al*, 1989; Wiesmuller *et al*, 1992), that is, molecularly defined adjuvants which are also known to stimulate the maturation of DCs via Toll-like receptor 2 (Hertz *et al*, 2001). Altogether, such liposomes are very appealing because they can combine, within a single construct, all the molecular elements that are necessary to elicit a highly effective antitumour immune response by channelling, *in vivo*, the tumour antigens into the DC-presentation pathways, and by provoking the differentiation of these cells into potent immunostimulatory APCs.

Here we report on the construction of monoepitope and diepitope liposomal vaccines designed to induce powerful, ErbB2-specific immune responses *in vivo.* As a Tc epitope, these constructs carry the ErbB2 peptide p63–71, which is efficiently presented by murine H-2K^d^ (Nagata *et al*, 1997). In addition, the diepitope construct contains a potent Th peptide derived from influenza virus haemagglutinin. Both peptides were coupled to the surface of the carrier vesicles via a synthetic Pam_3_CSS lipopeptide anchor. The ability of the liposomal vaccines to induce ErbB2-specific CTL responses and their antitumoral effects were investigated in immunocompetent BALB/c mice, using ErbB2-expressing murine renal carcinoma cells as a model.

## MATERIALS AND METHODS

### Lipids and peptides

Egg yolk L-*α*-phosphatidylcholine (PC), L-*α*-phosphatidyl-DL-glycerol (PG) transesterified from egg yolk PC and cholesterol (Chol) were purchased from Sigma-Aldrich (Saint Quentin Fallavier, France), and their purities (over 99%) were assessed by thin-layer chromatography. The lipopeptide Pam_3_CSS, prepared as previously described (Fernandes *et al*, 1997), was converted into the maleimide derivative Pam_3_CSS-maleimide (Pam_3_CSS-Mal) by reaction with 1-(2-aminoethyl)-pyrrole-2,5-dione according to Boeckler *et al* (1998). The two peptides ErbB2 p63–71 (CG-TYLPTNASL) and influenza virus haemagglutinin-derived HA307–319 (PKYVKQNTLKLAT-C) were obtained from Neosystem (Strasbourg, France). The cysteine or cysteinyl-glycine residues added to the C- or N-terminus of the peptides allow their facile coupling on the lipopeptide maleimide function. The purity of the peptides, as assessed by HPLC, was at least 80%.

### Preparation of liposomes

Liposomes were prepared by mixing phospholipids (PC, PG) and cholesterol, in a 75/20/50 molar ratio, in chloroform with the thiol-reactive functionalised lipopeptide Pam_3_CSS-Mal at 5 mol%, in a round-bottom flask (Boeckler *et al*, 1999). After solvent evaporation under high vacuum, the dried lipid film was hydrated in 1 ml 10 mM Hepes buffer (pH 6.5) containing 5% (w v^−1^) sorbitol by vortex mixing (1 ml for 10 *μ*mol lipids). The suspension was sonicated at 25°C (i.e. above the phase transition temperature of the lipids) for 1 h (5 s cycles interrupted for 1.25 s) under a continuous flow of nitrogen, using a 3 mm diameter probe sonicator (Vibra Cell, Sonics and Material Inc., Danbury, CT, USA) at 300 W. The small unilamellar vesicles preparation was finally centrifuged for 10 min at 10 000 **g** to remove the titanium dust originating from the probe.

### Peptide conjugation

ErbB2 and HA peptides were coupled by reacting freshly prepared liposomes containing Pam_3_CSS-Mal with the ErbB2 peptide alone (0.5 molar eq. of peptide *vs* surface accessible thiol-reactive maleimide function), or with the two peptides (ErbB2 and HA) in equimolar quantities (0.5 molar eq. of each peptide *vs* surface accessible thiol-reactive maleimide function). Coupling was performed, under argon, in 10 mM Hepes buffer (pH 6.5) containing 5% (w v^−1^) sorbitol, after reduction of the disulphide bonds of oxidised peptides with tris(2-carboxyethyl)phosphine (Sigma-Aldrich, Saint Quentin Fallavier, France) (0.7 eq. *vs* peptide). After 2 h at 25°C, a 10-fold excess of 2-mercaptoethanol was added to the preparation to derivatise all unreacted maleimide groups. This step was performed for 1 h under argon. Then, the liposomal preparation was dialysed extensively against 10 mM Hepes buffer (pH 7.4) containing 5% (w v^−1^) sorbitol, to eliminate unconjugated peptides and excess reagents. The phosphorous content of liposomes was analysed by a previously described method (Rouser *et al*, 1970).

Quantification of the peptides associated to vesicles was achieved by acid hydrolysis of the liposomal formulations (Boeckler *et al*, 1999). The amino acids that were generated were measured using a fluorimetric assay with fluorescamine (Böhlen *et al*, 1973) as follows: 50 *μ*l of the hydrolysis solution (containing 10–50 nmol of amines) was added to 1.5 ml of 50 mM sodium borate buffer (pH 9.0), followed by the addition of 500 *μ*l of a fluorescamine solution in dioxane (300 *μ*g ml^−1^). After mixing, the fluorescence was read immediately at *λ*_em_=475 nm (*λ*_exc_=390 nm). The contribution of the liposomes alone was evaluated using the same hydrolysis conditions, and subtracted from the total fluorescence. Hydrolysis of known quantities of free peptides was used as standard. The coupling yields were calculated *vs* the quantity of surface-exposed maleimide functions. The liposomal preparations were then concentrated using a Centricon type YM-100 (Millipore Corporation, Bedford, MA, USA) until a concentration of about 15 *μ*g ErbB2 peptide per 100 *μ*l was reached for injections into mice. The vesicle size was measured as described below, prior and after the conjugation of the peptide. Liposomes were finally frozen in liquid nitrogen after addition of a cryoprotectant (5% glucose) and stored at −20°C until use.

### Light-scattering measurements

Liposome size was determined by dynamic light scattering using a Zeta-master 3000 (Malvern instruments, Paris, France) with the following specifications: sampling time, 30 s; medium viscosity, 1.014 cP; refractive index, 1.34; scattering angle, 90°; temperature, 25°C. Data were analysed using the multimodal number distribution software included with the instrument. All the formed vesicles were uniformly distributed in size and exhibited average diameters of 65±15 nm, which, under our experimental conditions, remained unaffected by the freezing process.

### Cell lines and cell culture conditions

Murine renal carcinoma (Renca) cells stably expressing *Escherichia coli β*-galactosidase (Renca-lacZ) were maintained in RPMI 1640 (BioWhittaker, Verviers, Belgium) supplemented with 10% FBS (PAA Laboratories, Cölbe, Germany), 2 mM glutamine, 100 U ml^−1^ penicillin, 100 *μ*g ml^−1^ streptomycin and 0.25 mg ml^−1^ Zeocin. Renca-lacZ/ErbB2 cells expressing human ErbB2 (Maurer-Gebhard *et al*, 1998) were cultured in the same medium in addition containing 0.5 mg ml^−1^ G418.

### Antibodies

Anti-CD16/CD32 mAb 2.4G2 was purchased from BD PharMingen (Heidelberg, Germany), horseradish peroxidase (HRP)-coupled anti-mouse IgG antibody from Sigma-Aldrich (Deisenhofen, Germany). Cy5-conjugated anti-CD8*α* mAb YTS169 and FITC-conjugated anti-IFN-*γ* mAb XMG1.2 were kindly provided by HW Mittrücker, Max-Planck-Institut für Infektionsbiologie, Berlin, Germany.

### Restimulation of splenocytes and evaluation of T-cell responses

Female BALB/c mice of 15–17 g body weight (Charles River, Sulzfeld, Germany) were vaccinated by subcutaneous (s.c.) injection of Tc-ErbB2 liposomes, Tc-ErbB2/Th-HA liposomes or peptide-free liposome carrier on days 0 and 14. Amounts of liposomal formulations injected were adjusted for each mouse to receive 15 *μ*g of coupled Tc-ErbB2 peptide per injection. Importantly, the doses of (phospho)lipids injected were equivalent for all groups. At 5 days after the last vaccination, mice were killed, spleens were removed and single-cell suspensions were obtained by scraping organ tissues through a stainless steel mesh, followed by erythrocyte lysis. For detection of IFN-*γ*-expressing cells by flow cytometry, 1 × 10^6^ resuspended splenocytes ml^−1^ were stimulated for 5 h at 37°C with 10 *μ*g ml^−1^ of H-2K^d^ restricted ErbB2 p63-71 peptide TYLPTNASL (Thermo Hybaid, Ulm, Germany). During the final 4 h of incubation, 10 *μ*g ml^−1^ Brefeldin A (Sigma-Aldrich) was added. Cells were washed and incubated for 10 min with 2 *μ*g ml^−1^ anti-CD16/CD32 (Fc-block™) and 10 *μ*g ml^−1^ rat serum (Sigma-Aldrich) to block unspecific binding. Then cells were stained with Cy5-conjugated anti-CD8*α* mAb for 30 min at 4°C. Subsequently, cells were washed with PBS, fixed with 4% paraformaldehyde in PBS for 20 min at RT, and permeabilised with PBS, 0.1% BSA, 0.5% Saponin (Sigma-Aldrich) in the presence of 2 *μ*g ml^−1^ anti-CD16/CD32 and 10 *μ*g ml^−1^ rat serum. Then FITC-conjugated anti-IFN-*γ* mAb was added for 30 min at RT, cells were washed with PBS, transferred to PBS containing 1% paraformaldehyde, and analysed using a FACSCalibur flow cytometer.

### Detection of peptide-specific serum antibodies

For detection of peptide-specific antibodies in murine sera by enzyme-linked immunosorbent assay (ELISA), approximately 50 *μ*l of peripheral blood was collected from the tail vein of vaccinated mice at day 21 after the first immunization. Coagulated blood was separated from the serum by centrifugation at 4000 r.p.m. in a tabletop centrifuge (Hettich, Tuttlingen, Germany). Ninety-six-well ELISA plates (Nunc, Wiesbaden, Germany) were coated overnight at 4°C with 5 *μ*g ml^−1^ of ErbB2 peptide dissolved in bicarbonate buffer (pH 9.6), washed with PBS, 0.05% Tween 20, and blocked with 2% BSA in PBS for 2 h at 37°C, before 1 : 100 dilutions of serum samples were added. After 2 h of incubation at 37°C, plates were washed and HRP-conjugated secondary anti-mouse IgG antibody (Sigma-Aldrich) was added for 1 h at 37°C. After a final washing step, bound antibody complexes were detected by incubation with HRP-substrate ABTS (Roche, Mannheim, Germany) for 20 min at RT in the dark, followed by analysis of the absorption at 405/650 nm in an ELISA reader.

### Protective and therapeutic vaccination

BALB/c mice were vaccinated by s.c. injection of Tc-ErbB2 or Tc-ErbB2/Th-HA liposomes in an amount corresponding to 15 *μ*g of coupled Tc-ErbB2 peptide on days 0 and 14. On day 20, mice were inoculated with Renca-lacZ/ErbB2 or Renca-lacZ tumour cells by s.c. injection of 5 × 10^6^ cells into each flank. At regular intervals, two perpendicular tumour diameters were measured with a caliper, and tumour volumes were calculated according to the formula: length × (width)^2^ × 0.4. For therapeutic vaccination, naïve BALB/c mice were first inoculated with Renca-lacZ/ErbB2 cells as described above. At day 2 after tumour cell injection, when tumours were palpable, animals were treated by s.c. injection of Tc-ErbB2 or Tc-ErbB2/Th-HA liposomes in an amount corresponding to 7.5 *μ*g of coupled Tc-ErbB2 peptide, or peptide-free liposome carrier into the vicinity of each tumour. Treatment was repeated on day 6 and tumour growth was followed as described above. To avoid suffering of the animals, in all cases mice were killed latest when tumour size reached 0.8 cm^3^.

Long-term protection was investigated by intravenous re-challenge of surviving animals with 5 × 10^5^ Renca-lacZ/ErbB2 tumour cells 70 days after initial tumour cell inoculation. Cells were suspended in 100 *μ*l PBS and injected into the lateral tail vein. After 28 days, before mice developed any apparent disease symptoms, they were killed, lungs were excised, and pulmonary tumour nodules were visualised by X-gal staining and counted as described previously (Maurer-Gebhard *et al*, 1998). All animal experiments had been approved by the Regierungspräsidium Darmstadt, Germany, as the responsible government committee, and were conducted in a manner consistent with the guidelines of the UKCCCR.

## RESULTS

### Preparation of liposomal vaccine constructs

**Figure 1:**
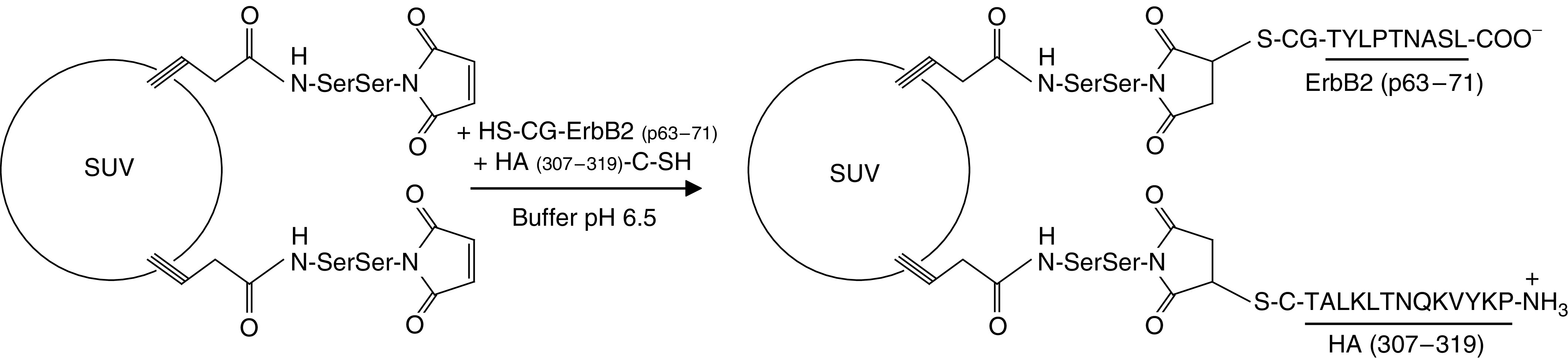
Design of the diepitope liposomal construct. Preformed negatively charged liposomes (PC/PG/Chol, 75/20/50 molar ratio; mean diameter: 65 nm) containing 5 mol% of the thiol-reactive lipopeptide anchor Pam_3_CSS-Mal were reacted, at 25°C and pH 6.5, with equimolar quantities of the peptides ErbB2 (p63–71), derivatised with a CG linker at its N-terminus, and HA307-319, derivatised with a C linker at its C-terminus.

Liposomal constructs were prepared by conjugation of the peptides TYLPTNASL (p63–71), derived from human ErbB2 (Nagata *et al*, 1997), and PKYVKQNTLKLAT (HA307–319), a promiscuous Th epitope derived from influenza virus haemagglutinin (HA) (O'Sullivan *et al*, 1991), to the surface of preformed small unilamellar vesicles under well-defined conditions that allow a chemoselective ligation of purified molecular species (Boeckler *et al*, 1999; Schelté *et al*, 2000) (Figure 1). The vesicles were obtained from egg phosphatidylcholine, phosphatidylglycerol and cholesterol in a 75/20/50 molar ratio, and the thiol-reactive maleimide-functionalised lipopeptide Pam_3_CSS-Mal (Boeckler *et al*, 1998) was added at a 5 mol% ratio. This amphiphatic lipopeptide, which serves as anchor for both Tc and Th epitopes, was selected because it is known to efficiently target exogenous antigens to the MHC class I/II-restricted presentation pathways in APCs (Deres *et al*, 1989; Bessler and Jung, 1992). Owing to the coupling strategy, the two peptides were functionalised with a thiol group via the introduction at their N- or C-terminus of a cysteinyl-glycine or a cysteine linker (see Materials and methods). The determination of peptides coupled to the surface of the liposomes showed a global yield of 90% for the diepitope construct (Tc-ErbB2/Th-HA liposomes) and 100% for the mono-epitope construct (Tc-ErbB2 liposomes). A uniform size distribution of vesicles was obtained with a mean diameter of 65±15 nm, which was unaffected by the coupling step.

### Protection of mice from challenge with ErbB2-expressing tumour cells

To investigate whether immunisation with liposomal vaccine constructs can induce antitumour immunity and protect animals from subsequent tumour challenge, we used a model that allows evaluation of ErbB2-specific reagents in immunocompetent mice. This model is based on BALB/c-derived renal carcinoma (Renca) cells, which form solid tumours or lung tumour nodules in BALB/c mice upon s.c. and i.v. injection, respectively (Murphy and Hrushesky, 1973). Importantly, it was shown previously that Renca cell variants which express *E. coli β*-galactosidase (Renca-lacZ), or coexpress *β*-galactosidase and human ErbB2 (Renca-lacZ/ErbB2), are not rejected by naïve immunocompetent animals (Maurer-Gebhard *et al*, 1998). Moreover, the H-2K^d^-restricted ErbB2 peptide TYLPTNASL (p63-71) serving as a Tc epitope in the liposomal vaccine formulations can be efficiently presented in BALB/c mice (Nagata *et al*, 1997).

**Figure 2:**
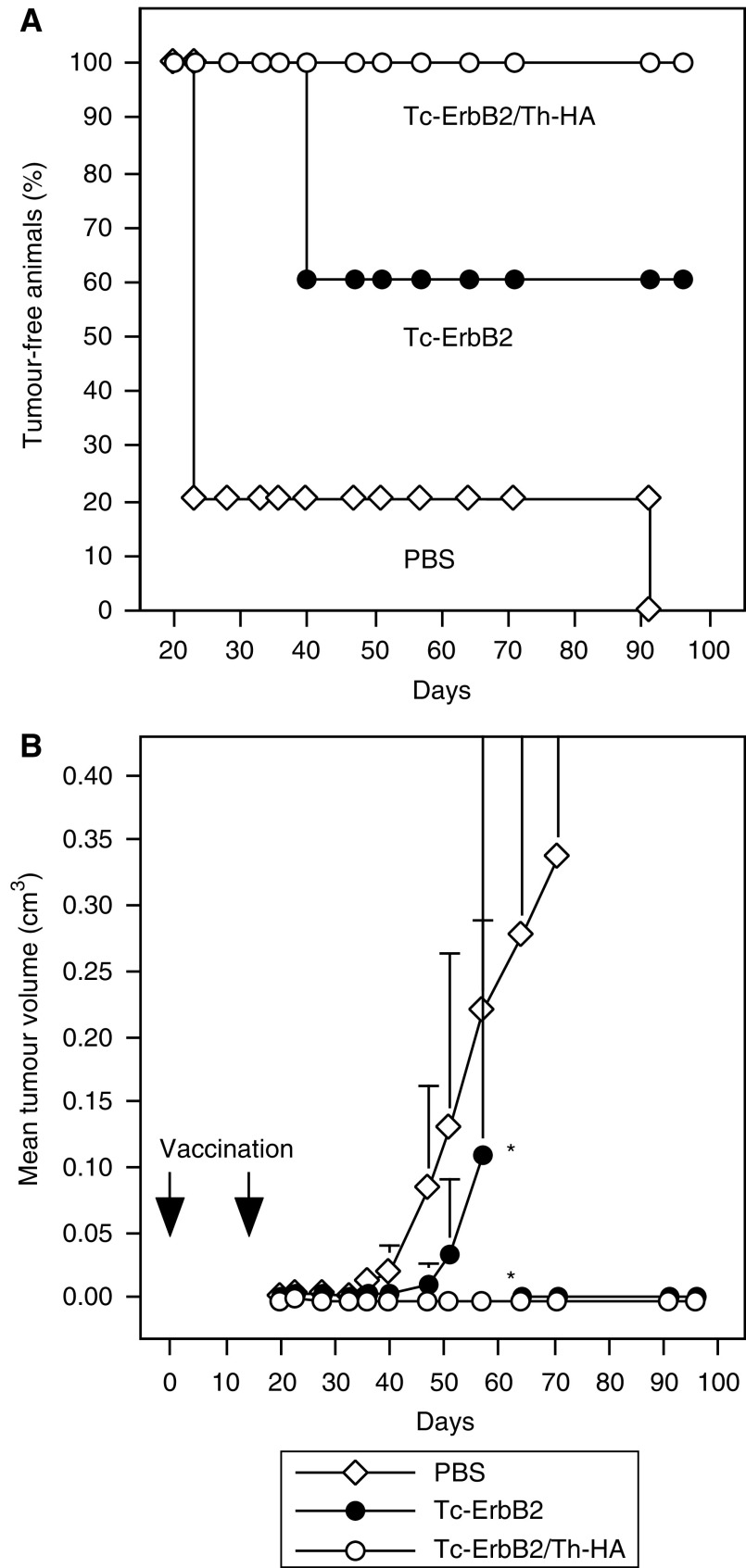
Protective vaccination of BALB/c mice. Five animals per group were immunised on days 0 and 14 by s.c. injection of Tc-ErbB2/Th-HA liposomes (○) or Tc-ErbB2 liposomes (•) corresponding to 15 *μ*g of coupled Tc-ErbB2 peptide per injection. Control animals received PBS (open diamonds). On day 20, mice were inoculated with Renca-lacZ/ErbB2 tumour cells by s.c. injection into each flank. Tumour growth was followed by caliper measurements and mean tumour volumes were calculated. (**A**) Tumour-free survival. Tumour growth was registered when palpable tumours had formed at one or both of the injected flanks. (**B**) Kinetics of tumour growth of the experiment shown in (**A**). As indicated by asterisks, two of the Tc-ErbB2 vaccinated animals had to be killed on day 57 due to tumour growth. From day 64 onwards, mean tumour volume in this group was calculated from the remaining three animals. Tumour-bearing mice were killed latest when tumour size reached 0.8 cm^3^, to avoid suffering of the animals.

Mice were vaccinated twice on days 0 and 14 by s.c. injection of Tc-ErbB2 or Tc-ErbB2/Th-HA liposomes, corresponding to 15 *μ*g of coupled Tc-ErbB2 peptide per injection. Control animals received PBS. This route of administration was chosen because the skin contains DCs previously demonstrated to actively promote CTL activity (Campton *et al*, 2000), and because after application liposomes distribute preferentially via the lymph reaching local organised lymphoid tissues (Oussoren and Storm, 2001). At 6 days after the last vaccination, mice were challenged by s.c. injection of Renca-lacZ/ErbB2 cells into each flank, and tumour growth was followed. Tumour-free survival of the animals is shown in Figure 2A, tumour growth kinetics are shown in Figure 2B. Whereas early onset of tumour growth was observed in the majority of the PBS-treated control animals, three out of five mice vaccinated with the Tc-ErbB2 construct rejected Renca-lacZ/ErbB2 tumour challenge and remained tumour free for the complete duration of the experiment until day 96. Vaccination with Tc-ErbB2/Th-HA liposomes was even more effective, resulting in complete protection of all five mice in this group from outgrowth of Renca-lacZ/ErbB2 tumours.

### Protection of vaccinated mice against repeated tumour challenge

To analyse whether mice vaccinated with liposomal vaccine preparations were protected against repeated tumour challenge, mice from the experiment described above that had rejected initial tumour challenge were re-challenged after day 96 by i.v. injection of Renca-lacZ/ErbB2 cells. In naïve animals, i.v. injection of Renca-lacZ cells results in the formation of pulmonary tumour nodules which can be detected upon X-gal staining of the organs (Maurer-Gebhard *et al*, 1998). As summarised in Table 1, in the control mice which had not been previously vaccinated and challenged, 4 weeks after i.v. injection of tumour cells more than 250 tumour nodules had developed on each lung surface. In contrast, all animals that had received Tc-ErbB2 or Tc-ErbB2/Th-HA liposomes as vaccines and had rejected initial s.c. Renca-lacZ/ErbB2 tumour challenge also remained completely free of pulmonary tumour nodules (Table 1). These results demonstrate that vaccination with Tc-ErbB2 or Tc-ErbB2/Th-HA liposomes is highly effective and can protect against repeated challenge with ErbB2-expressing tumour cells.

### Specificity of tumour cell rejection

**Figure 3:**
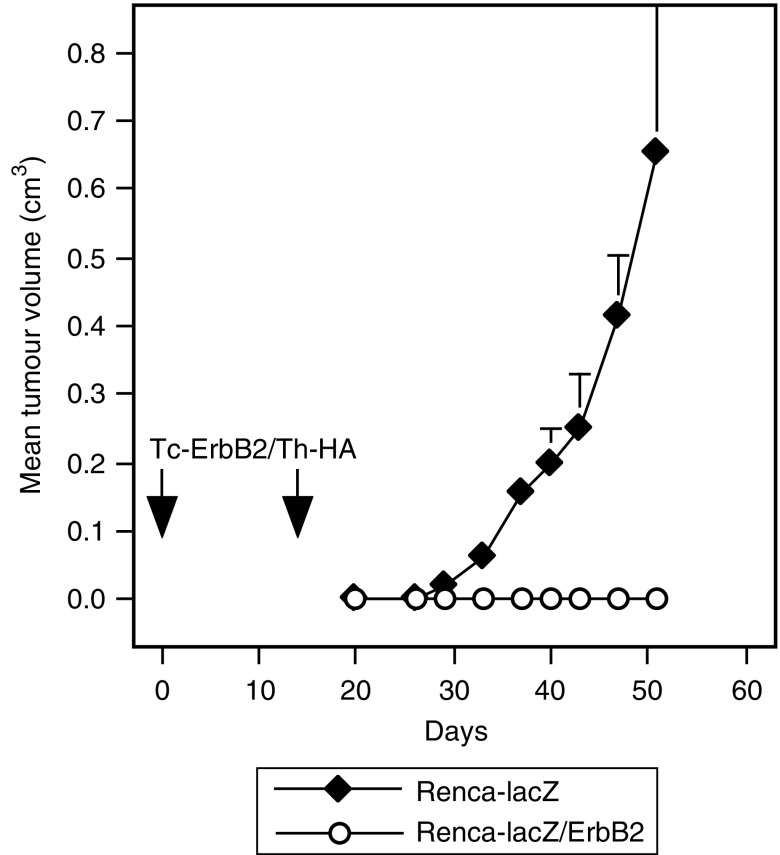
Specificity of tumour rejection. Two groups of BALB/c mice were immunised on days 0 and 14 by s.c. injection of Tc-ErbB2/Th-HA liposomes corresponding to 15 *μ*g of coupled Tc-ErbB2 peptide per injection. On day 20, one group of animals was inoculated with Renca-lacZ/ErbB2 tumour cells (open circles) by s.c. injection into each flank. The other group was injected with ErbB2-negative parental Renca-lacZ cells (filled diamonds). Tumour growth was followed by caliper measurements and mean tumour volumes were calculated. Tumour-bearing mice were killed latest when tumour size reached 0.8 cm^3^, to avoid suffering of the animals.

To test whether antitumoural responses resulting from vaccination with liposomal constructs were dependent on the expression of ErbB2 by the tumour cells, two groups of mice were vaccinated twice on days 0 and 14 by s.c. injection of Tc-ErbB2/Th-HA liposomes, corresponding to 15 *μ*g of coupled Tc-ErbB2 peptide per injection. At 6 days after the last vaccination, animals from one group were challenged by s.c. injection of Renca-lacZ/ErbB2 cells into each flank as described above, the other group was injected with parental ErbB2-negative Renca-lacZ cells. Tumour growth kinetics are shown in Figure 3. Whereas all mice vaccinated with Tc-ErbB2/Th-HA liposomes and challenged with ErbB2-expressing cells remained tumour free, none of the Tc-ErbB2/Th-HA vaccinated animals were able to reject challenge with ErbB2 negative but otherwise isogenic tumour cells. These results confirm that liposomal ErbB2 peptide vaccines induce immune responses specifically directed against ErbB2-expressing tumours.

### Induction of ErbB2-specific T-lymphocytes by liposomal vaccine constructs

**Figure 4:**
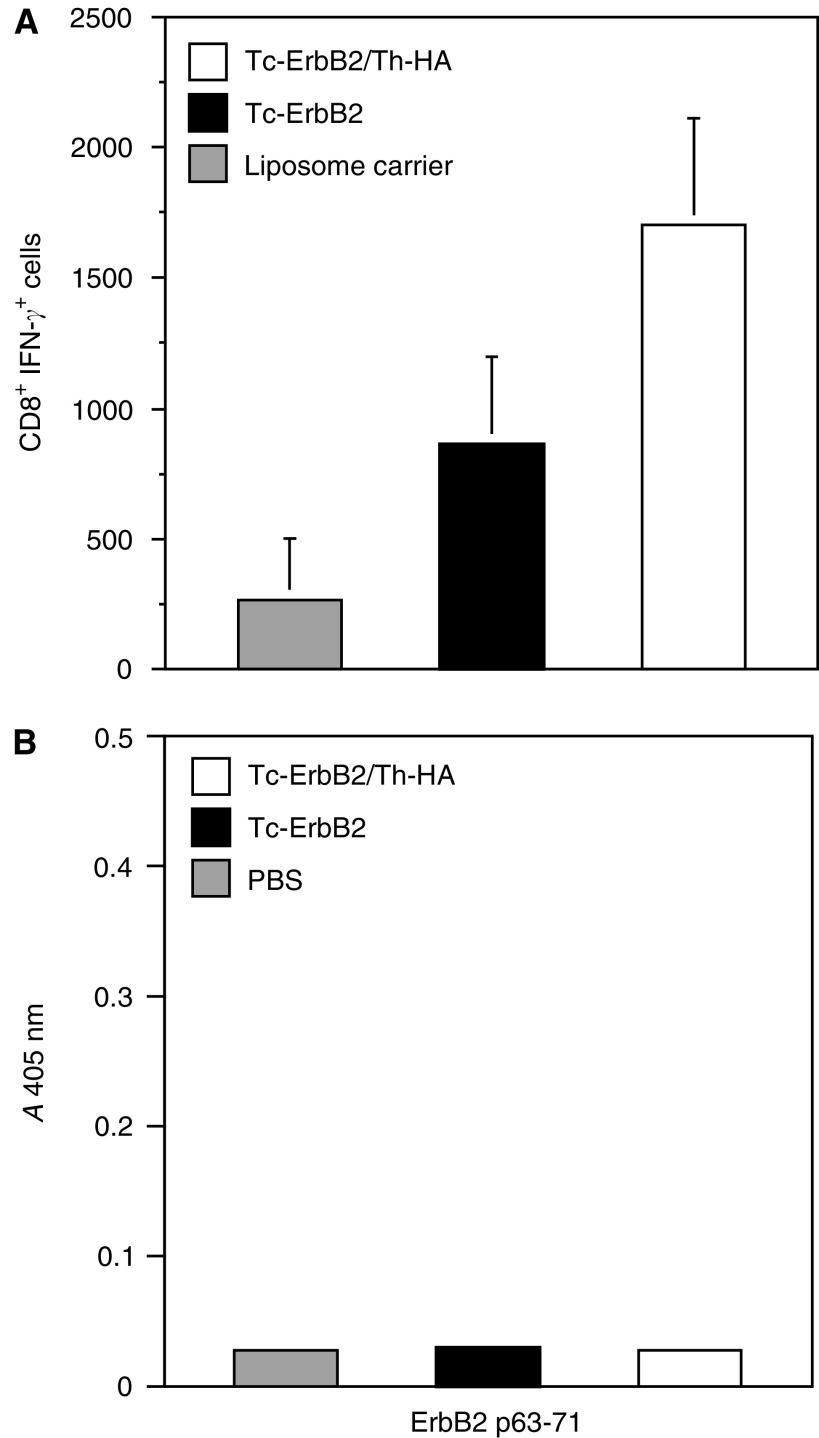
Evaluation of vaccine-induced immune responses. BALB/c mice were immunised in separate experiments by s.c. injection of Tc-ErbB2/Th-HA liposomes (white bars), Tc-ErbB2 liposomes (black bars), or peptide-free liposome carrier (**A**) or PBS (**B**) (hatched bars) on days 0 and 14. (**A**) To investigate induction of ErbB2-specific CTLs, 5 days after vaccination splenocytes were isolated and stimulated with synthetic ErbB2 peptide before flow cytometric analysis with anti-CD8*α* and anti-IFN-*γ* antibodies. Absolute numbers of CD8^+^ IFN-*γ*^+^ splenocytes are indicated (mean values from five animals per group). (**B**) Sera taken at day 21 after the first vaccination were tested for the presence of peptide epitope-specific antibodies by ELISA. ErbB2-derived Tc peptide was coated on ELISA plates, before sera were added. Peptide-specific antibodies were detected by HRP-conjugated secondary anti-mouse IgG, followed by addition of HRP-substrate (ABTS) and measuring of the absorbance at 405 nm in an ELISA reader.

To analyse the nature of the immune responses induced by the liposomal vaccines, BALB/c mice were immunised twice by s.c. injection of Tc-ErbB2 liposomes, Tc-ErbB2/Th-HA liposomes, or peptide-free liposomal carrier as a control at days 0 and 14. Amounts of liposomal formulations injected were adjusted for each mouse to receive 15 *μ*g of coupled Tc-ErbB2 peptide per injection. At 5 days after the last vaccination, mice were killed, splenocytes were isolated, and restimulated ex *vivo* with ErbB2-derived synthetic peptide TYLPTNASL for 5 h. Activated CD8^+^ T cells were identified by flow cytometry after double-staining of splenocytes with antibodies detecting CD8 and intracellular IFN-*γ*. The results shown in Figure 4A demonstrate that immunisation of mice with both Tc-ErbB2 and Tc-ErbB2/Th-HA liposomes induced activation of CD8^+^ T cells specific for the Tc-ErbB2 epitope. Importantly, the diepitope construct Tc-ErbB2/Th-HA was more potent, resulting in a doubling of the absolute numbers of ErbB2-specific CD8^+^ IFN-*γ*^+^ T cells when compared to mono-epitope Tc-ErbB2 liposomes. In contrast, peptide-free liposomal carrier did not lead to an increase of ErbB2-specific effector cells, with numbers of CD8^+^ IFN-*γ*^+^ T cells in this group comparable to those of untreated animals (data not shown).

Potential antibody responses to the Tc-ErbB2 peptide upon vaccination with peptide-carrying liposomes were also investigated. At day 21 after the first immunisation, sera were collected from BALB/c mice injected twice with Tc-ErbB2 liposomes or Tc-ErbB2/Th-HA liposomes as described above, and were tested for the presence of peptide-specific antibodies by ELISA. Sera from PBS-treated animals served as a control. As shown in Figure 4B, none of the mice immunised with Tc-ErbB2 or Tc-ErbB2/Th-HA liposomes developed antibodies reacting with the Tc-ErbB2 peptide. Taken together, these results indicate that the antitumoural activity of the Tc-ErbB2 peptide carrying liposomal constructs is mediated by a strong, ErbB2-specific T-cell response induced by the vaccines. Humoral responses did not play a role in the observed tumour rejection.

### Therapeutic vaccination of tumour-bearing animals

**Figure 5:**
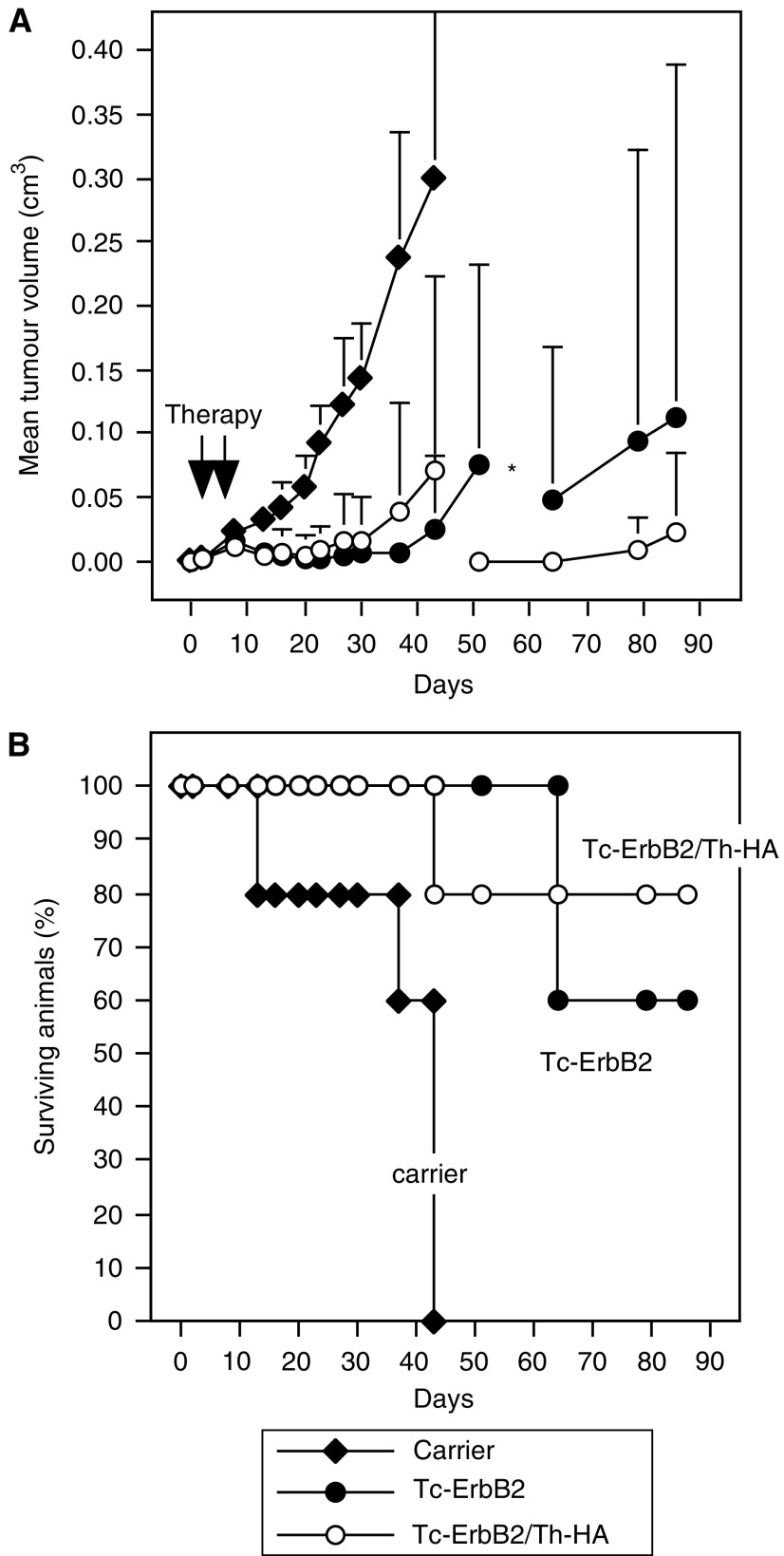
Therapeutic vaccination of tumour-bearing animals. BALB/c mice were inoculated with Renca-lacZ/ErbB2 tumour cells by s.c. injection into each flank. When tumours were palpable, animals were treated twice by s.c. injection of Tc-ErbB2/Th-HA liposomes (○), Tc-ErbB2 liposomes (•), or liposomal carrier as a control (⧫) into the vicinity of each tumour. (**A**) Tumour growth was followed by caliper measurements and mean tumour volumes were calculated. As indicated by the asterisk, due to tumour growth one of the Tc-ErbB2/Th-HA- and two of the Tc-ErbB2-treated animals had to be killed on days 43 and 51, respectively. Subsequently, mean tumour volumes in these groups were calculated from the remaining animals. In the control group, all mice were killed due to tumour growth latest until day 43. (**B**) Course of the disease in mice from the experiment shown in (**A**). In addition to the animals that had completely rejected their tumours and were tumour free, in each group treated with Tc-ErbB2/Th-HA or Tc-ErbB2 liposomes one animal developed only small tumours until day 86 when the experiment was terminated. All other tumour-bearing mice were killed latest when tumour size reached 0.8 cm^3^ to avoid suffering of the animals.

The influence of liposomal vaccine constructs on the growth of established tumours was investigated in animals carrying Renca-lacZ/ErbB2 tumours. BALB/c mice were inoculated with tumour cells by s.c. injection into each flank. Upon formation of palpable tumours (day 2 post tumour cell injection), animals were treated by s.c. injection of Tc-ErbB2 or Tc-ErbB2/Th-HA liposomes corresponding to 7.5 *μ*g of coupled ErbB2 Tc peptide into the vicinity of each tumour. Control animals received peptide-free liposomal carrier. Treatment was repeated 4 days later, and tumour growth was followed. The results are shown in Figure 5. In most vaccinated mice, tumour growth continued for a few days after onset of therapy, before therapeutic effects became apparent (Figure 5A).

Whereas injection of liposomal carrier had no effect on tumour growth, and all animals in this group had to be killed due to the size of their tumours latest until day 43 of the experiment (Figure 5B), two out of five mice treated by therapeutic vaccination with mono-epitope Tc-ErbB2 liposomes and three out of five mice treated with diepitope Tc-ErbB2/Th-HA liposomes had completely rejected the established tumours by day 20 and remained tumour free until day 86 when the experiment was terminated. In the remaining mice of these groups, vaccination resulted in delayed tumour growth in comparison to the control animals. In addition to the tumour-free mice, one animal in each group that had received ErbB2 vaccines developed only small tumours and therefore did not have to be killed before the end of the experiment (Figure 5B).

## DISCUSSION

The aim of the present study was the design of cancer vaccines composed of well-characterised synthetic molecules that cooperate in the activation of a powerful cell-mediated antitumour immune response. We have used liposomal carriers as multivalent vectors for Tc and Th peptide epitopes which are covalently linked to the surface of the vesicles via Pam_3_CSS, a specific lipopeptide anchor previously shown to have powerful immunostimulatory activity (Bessler and Jung, 1992). For the coupling step we made use of a strategy we have developed previously (Boeckler *et al*, 1999). Accordingly, a synthetic lipopeptide derivative such as Pam_3_CSS-Mal, which contains a thiol-reactive maleimide function (Boeckler *et al*, 1998), was first incorporated into the bilayers of small unilamellar vesicles (mean dia. 65 nm) masking the hydrophobic nature of the anchor/adjuvant. In the subsequent steps, the peptide epitopes that carry a free thiol function either at their N- or C-terminus are conjugated to the surface of these preformed and functionalised vesicles. This *a posteriori* coupling of peptides to lipopeptides is thus achieved under very mild conditions in aqueous media via a high-yield chemoselective ligation. For the Tc-ErbB2 liposomal construct, the ErbB2 epitope p63–71 was used. This peptide can be presented by human HLA-A2402 (Okugawa *et al*, 2000), but it has also been shown to be efficiently presented as an immunodominant CTL epitope by murine H-2K^d^ in BALB/c mice (Nagata *et al*, 1997). Previously, co-delivery of this peptide with immunostimulatory CpG ODN encapsulated within liposomes has been demonstrated to induce ErbB2-specific CTL responses in BALB/c mice (Li *et al*, 2003). However, potential antitumoral activity of such vaccines *in vivo* was not investigated.

Using BALB/c-derived renal carcinoma (Renca) cells as a model system, we could show in the present study that the mono-epitope liposomes carrying the Tc-ErbB2 peptide induced protective cellular immunity against tumour cells expressing human ErbB2, resulting in the rejection of s.c. implanted Renca-lacZ/ErbB2 cells in the majority of the vaccinated animals. Thereby immunological memory was induced, leading to long-term systemic immunity in the tumour-free animals and complete protection, several months later, against subsequent re-challenge with intravenously injected Renca-lacZ/ErbB2 cells. In the second vaccine formulation (Tc-ErbB2/Th-HA liposomes), in addition to the ErbB2 Tc epitope, a Th epitope derived from influenza haemagglutinin (HA307-319) was also coupled to the liposomal carriers. This Th epitope was chosen because of its promiscuous binding to MHC class II molecules and recognition by T cells (O'Sullivan *et al*, 1991). The diepitope liposomal construct proved to be even more potent as a cancer vaccine. It protected all vaccinated mice from subsequent s.c. challenge and i.v. re-challenge with ErbB2-expressing Renca-lacZ/ErbB2 cells. Importantly, the immune responses initiated were specifically directed to the ErbB2 antigen. Thus, vaccinated mice were protected against challenge with Renca-lacZ/ErbB2, but not against ErbB2-negative Renca-lacZ cells still expressing bacterial *β*-galactosidase, which in naïve animals in our Balb/c-based model fails to serve as a tumour rejection antigen (Maurer-Gebhard *et al*, 1998). This demonstrates that the potent antitumoral responses observed were not caused solely by broad, unspecific immunostimulatory activity of the vaccines.

The combination of Tc and Th epitopes in the Tc-ErbB2/Th-HA liposomes was primarily intended to enhance the function of DCs and improve activation of CD8^+^ T-cells through the recruitment of CD4^+^ T-lymphocytes. Although proper activation of DCs for induction of CTL responses can be achieved by both CD4^+^-dependent and independent pathways (Schuurhuis *et al*, 2000), the help of Th epitopes and CD4^+^ T-lymphocytes has been shown to be crucial in optimising CTL responses and for efficient and long-lasting immunity against tumours (Toes *et al*, 1999). Activation and maturation of APCs are dependent on licensing signals, one of which can be provided by activated helper T cells via ligation of CD40. Then upon CD40 ligation, APCs become ‘licensed’ to prime CTLs, due to the interaction between CD40 receptor expressed on the DC and CD40 ligand present on the Th cells, triggering the production of IL-12 and initiating the CTL response (Koch *et al*, 1996; Schoenberger *et al*, 1998). Indeed, in our study addition of the highly immunogenic Th epitope clearly increased the efficiency of Tc-ErbB2 liposomes to stimulate T cells specific for the ErbB2-derived Tc epitope. This is most likely due to helper T cells activated by DCs presenting the Th epitope. In turn, such helper T cells could then provide licensing signals towards DCs, thereby increasing presentation of the Tc epitope and activation of ErbB2-specific CTLs. Of note, the critical role of helper T cells for the activation of tumour-specific CTLs has also become apparent in recent studies with regular ErbB2 peptide vaccines. Vaccination of cancer patients with a single ErbB2-derived CTL epitope together with GM-CSF as an adjuvant was not very effective, leading only to short-lived CTL responses (Knutson *et al*, 2002). In contrast, vaccines consisting of different potential ErbB2 Th epitopes also encompassing a number of HLA-A2 motifs induced long-lived CD4^+^ and CD8^+^ T-cell responses (Knutson *et al*, 2001). However, so far this has not resulted in clinical responses.

The Th/Tc collaboration in the induction of a CTL response could be corroborated by the observation that for each liposomal construct, results from ex *vivo* restimulation assays and protective vaccination experiments corresponded well. Indeed, vaccination of BALB/c mice with either Tc-ErbB2-liposomes or Tc-ErbB2/Th-HA liposomes induced activation of CD8^+^ T cells specific for the ErbB2-derived Tc epitope TYLPTNASL, as demonstrated by intracellular IFN-*γ* staining. However, in mice vaccinated with the diepitope Tc-ErbB2/Th-HA liposomes, a two times higher number of CD8^+^ T cells, most likely CTL, was activated in comparison to animals that had received mono-epitope Tc-ErbB2 liposomes. A marked enhancement of antitumoral activity of liposomes containing Tc and Th peptides was also observed upon therapeutic application of the liposomal formulations in tumour-bearing animals. The induction of ErbB2-specific CD8^+^ T cells clearly indicates that the tested liposomal vaccines activate T-cell-mediated, antigen-specific antitumour immunity. ErbB2-specific humoral responses were not induced. Consequently, anti-ErbB2 antibodies were not involved in tumour rejection.

As a tumour antigen, here we have used human ErbB2, which is a foreign antigen in this system despite a high degree of sequence identity with its murine counterpart and the fact that Renca-lacZ/ErbB2 cells are not rejected in naïve immunocompetent animals. However, CTLs from Balb/c mice that similar to the CD8^+^ T cells identified in our study, recognise the human ErbB2 epitope TYLPTNASL have previously been shown to also lyse cells presenting the corresponding murine ErbB2 peptide TYLPANASL (Nagata *et al*, 1997). This indicates that cross-reactive immune responses are possible, and suggests that at least for this particular epitope tolerance to endogenous murine ErbB2 might have to be overcome to mount an effective response against the human peptide. Subsequent studies in animals such as the recently described ErbB2/HER2 transgenic mice (Piechocki *et al*, 2003) will help to further investigate the potential of liposomal ErbB2 vaccines to break endogenous tolerance against this antigen.

As mentioned above, in the liposomal vaccine constructs investigated in our study, both Tc and Th peptide epitopes were conjugated to Pam_3_CSS, a synthetic analogue of the N-terminus of *E. coli* lipoprotein. The choice of this lipopeptide anchor is highly important for the immune response, since it allows the targeting, via poorly understood mechanisms (Uhl *et al*, 1991; Andrieu *et al*, 2003), of exogenous antigens into MHC class I and class II restricted pathways. Lipopeptides, such as Pam_3_CSS, constitute potent and nontoxic immunoadjuvants which, for example, activate B lymphocytes (Bessler *et al*, 1985) and APCs such as macrophages and DCs (Hertz *et al*, 2001). Moreover, *in vivo*, they are able to induce cell-mediated as well as humoral immune responses, depending on the nature of the epitope, against low molecular weight peptides that are covalently coupled to them (Deres *et al*, 1989; Wiesmuller *et al*, 1992). This might explain why liposomes only containing the lipopeptide-coupled Tc epitope were also able to induce significant ErbB2-specific immunity and also why, in the case of Tc-ErbB2/Th-HA liposomes, an additional help from CD4^+^ T cells could be observed. Importantly, in contrast to lipidated (e.g. palmitoylated) peptides, inclusion of the Pam_3_CSS lipopeptide anchor in the liposomal formulations might also induce Toll-like receptor 2-mediated internalisation by DCs (Hertz *et al*, 2001). Immature DCs could thereby become activated and upregulate costimulatory molecules and MHC class II expression.

In conclusion, our data demonstrate that effective priming of ErbB2-specific CD8^+^ T cells occurred upon *in vivo* application of cancer vaccines consisting of an ErbB2-derived Tc-epitope attached to a liposomal carrier via an adjuvant Pam_3_CSS lipopeptide anchor. The importance of CD4^+^ T cells for the resulting immune responses could not be analysed in detail. However, upon administration of a liposomal formulation that in addition to the Tc epitope also included an influenza Th epitope, improved effector activities were observed. Both protective and therapeutic vaccination with this diepitope Tc-ErbB2/Th-HA liposomal construct induced stronger antitumour immunity than vaccination with mono-epitope Tc-ErbB2 liposomes, accompanied by the recruitment of a higher number of IFN-*γ*-producing T cells specific for the ErbB2 peptide TYLPTNASL. This strongly supports a beneficial role of T-cell help in the induction of antitumour immune responses. Therefore, the liposomal cancer vaccines investigated in this study that combine Tc- and strong Th-epitopes fused to an immunostimulatory lipopeptide anchor might provide a means to increase immunogenicity and enhance the antitumoural activity of synthetic peptide vaccines. Such novel liposomal vaccines might improve the efficiency of common peptide vaccination strategies using individual peptides that have been defined as potential tumour rejection antigens.

## Figures and Tables

**Table 1:** Long-term protection of vaccinated animals

**Vaccination**	**Mean number of pulmonary tumour nodules**
Tc-ErbB2/Th-HA	0 (*n*=*5*)
Tc-ErbB2	0 (*n*=*2*)
None	>250 (*n*=*5*)

After rejection of initial tumour challenge, tumour-free mice from the experiment shown in Figure 2 were re-challenged by i.v. injection of Renca-lacZ/ErbB2 cells after day 96. After 28 days, before mice developed any apparent disease symptoms, they were killed, lungs were excised, and pulmonary tumour nodules were visualised by X-gal staining of excised organs and counted. Naïve animals injected with Renca-lacZ/ErbB2 cells served as a control. One of the three mice in the Tc-ErbB2 vaccinated group that had rejected the initial tumour challenge died for unknown reasons before i.v. re-challenge could be performed.
